# Maximizing efficiency of rumen microbial protein production

**DOI:** 10.3389/fmicb.2015.00465

**Published:** 2015-05-15

**Authors:** Timothy J. Hackmann, Jeffrey L. Firkins

**Affiliations:** ^1^Department of Animal Sciences, University of FloridaGainesville, FL, USA; ^2^Department of Animal Sciences, The Ohio State UniversityColumbus, OH, USA

**Keywords:** rumen microbiology, reserve carbohydrate, glycogen, energy spilling, short-chain fatty acids

## Abstract

Rumen microbes produce cellular protein inefficiently partly because they do not direct all ATP toward growth. They direct some ATP toward maintenance functions, as long-recognized, but they also direct ATP toward reserve carbohydrate synthesis and energy spilling (futile cycles that dissipate heat). Rumen microbes expend ATP by vacillating between (1) accumulation of reserve carbohydrate after feeding (during carbohydrate excess) and (2) mobilization of that carbohydrate thereafter (during carbohydrate limitation). Protozoa account for most accumulation of reserve carbohydrate, and in competition experiments, protozoa accumulated nearly 35-fold more reserve carbohydrate than bacteria. Some pure cultures of bacteria spill energy, but only recently have mixed rumen communities been recognized as capable of the same. When these communities were dosed glucose *in vitro*, energy spilling could account for nearly 40% of heat production. We suspect that cycling of glycogen (a major reserve carbohydrate) is a major mechanism of spilling; such cycling has already been observed in single-species cultures of protozoa and bacteria. Interconversions of short-chain fatty acids (SCFA) may also expend ATP and depress efficiency of microbial protein production. These interconversions may involve extensive cycling of intermediates, such as cycling of acetate during butyrate production in certain butyrivibrios. We speculate this cycling may expend ATP directly or indirectly. By further quantifying the impact of reserve carbohydrate accumulation, energy spilling, and SCFA interconversions on growth efficiency, we can improve prediction of microbial protein production and guide efforts to improve efficiency of microbial protein production in the rumen.

## Introduction

Cattle and other ruminants can degrade fibrous feedstuffs owing to the consortium of bacteria, protozoa, fungi, and methanogens inhabiting their rumen and hindgut. This consortium ferments fiber and other feed components to short-chain fatty acids (SCFA) and, in the process, generates ATP that fuels microbial growth (synthesis of cellular protein in particular). This microbial protein supplies 60 to 85% of amino acids (AA) reaching the animal's small intestine (Storm et al., 1983). Maximizing efficiency of its production would consequently improve cattle productivity.

Production of microbial protein is inefficient because microbes do not direct all ATP toward growth. Rather, microbes can direct some ATP toward maintenance functions, synthesis of reserve carbohydrate, or energy spilling (futile cycles that dissipate heat). The impact of maintenance functions on growth efficiency has been recognized for decades and for both pure and mixed cultures (Pirt, 1965; Russell and Cook, 1995). Only recently, however, have reserve carbohydrate accumulation and energy spilling been accurately quantified in mixed rumen microbes such that their impact on efficiency can be considered (Hackmann et al., 2013a,b; Denton et al., 2015). We will review these recent advances in the context of other factors that depress efficiency of microbial protein production.

For many years, knowledge on the rumen microbiome steadily grew but was viewed myopically—from the context of a relatively few culturable isolates. Researchers used pure cultures or mixtures of a few pure cultures to establish niches, substrates used, growth factors, growth rates, fermentation end-products, and fundamental interactions between those cultures (Krause et al., 2013). Results from mixed cultures and from rumen-cannulated animals expanded those principles. In the past decade, however, the expansion of technology has greatly expanded our view of the rumen microbiome, sometimes from a hyperopic view—from the context of how to integrate extensive metagenomics data on the rumen microbiome with ruminant nutrition (Firkins and Yu, 2015). We will discuss how the growth efficiency of mixed ruminal microbes is affected by metabolic fluxes of anabolic and catabolic reactions, with emphasis on energy spilling.

## Improving efficiency of microbial growth

For more than 40 years, we have recognized that microbes grow (synthesize microbial protein) with far from perfect efficiency (Stouthamer, 1973). For mixed rumen microbes *in vivo*, actual growth efficiency ranges from only 1/3 to 2/3 of the theoretical maximum (as calculated from biochemical pathways) (Table 1). This implies that microbes spend as little as 1/3 of ATP on growth. Similar efficiencies are reported for mixed and pure cultures of bacteria *in vitro* (Table 1).

**Table 1:** **Efficiency of rumen microbial growth**.

**Organism**	**Efficiency**	
	**g microbial DM (mol ATP)^−1^^a^**	**% of theoretical maximum^b^**
Mixed rumen microbes, *in vivo*	11–21	34–66
Mixed rumen bacteria, *in vitro*	7.5–16.7	23–52
Pure cultures, *in vitro*	10–25	31–78

a *Summarized from Russell and Wallace (1997)*.

b *31.9 g (g microbial DM mol ATP)^−1^; value from Stouthamer (1973) for growth with glucose, amino acids, and nucleic acid bases*.

**Figure 1:**
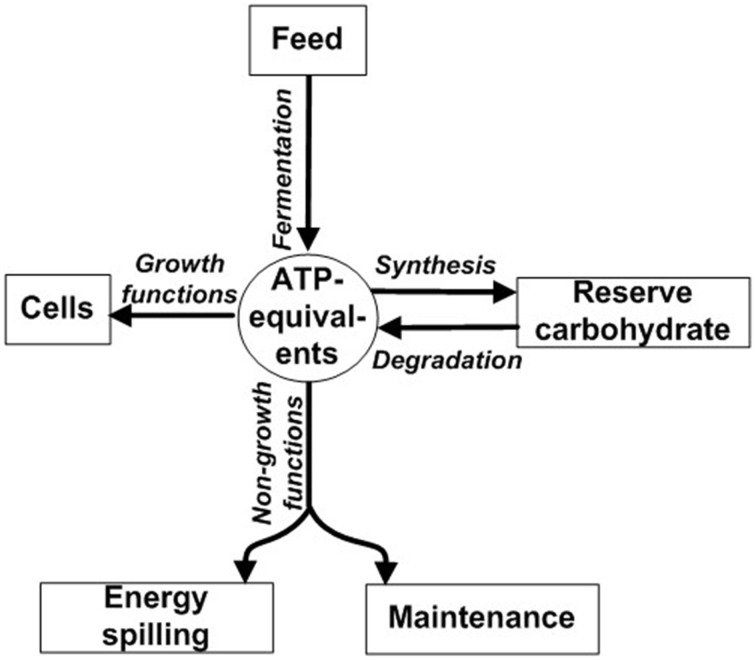
**Partitioning of ATP energy toward growth functions, non-growth functions, and synthesis of reserve carbohydrate**. ATP-equivalents can include ATP or ATP-yielding carbon compound (e.g., glucose). Modified from Russell and Wallace (1997) and Russell (2007a).

ATP not spent on growth is instead directed toward non-growth functions such as maintenance, energy spilling, and synthesis of reserve carbohydrate (Figure 1). Maintenance functions are those required for cellular “housekeeping” and include (1) re-synthesis of protein following intracellular turnover and (2) maintaining ion balances across the cell membrane (Russell and Cook, 1995). Motility is also a component of maintenance; it is a special case of maintaining ion balances because motility is driven by a proton or sodium motive force (Russell and Cook, 1995). Reserve carbohydrate synthesis refers to formation of glycogen and other compounds during energy excess (Preiss and Romeo, 1989). Although reserve carbohydrate can be mobilized later for growth (Figure 1) (Wilkinson, 1959), some ATP is irreversibly expended during synthesis. Energy spilling (e.g., futile cycling of ions or reserve carbohydrate; Russell and Cook, 1995; Portais and Delort, 2002; Russell, 2007b) refers to energy dissipated as heat when ATP exceeds needs for growth, maintenance functions, and reserve carbohydrate synthesis. It can be analogized to water spilling over the brim of an overfilled bucket (Figure S1). It is commonly a response to excess carbohydrate (Russell, 1998), as would occur when the ruminant is fed grain.

Maintenance functions have been long-recognized to be a sink for ATP energy and responsible for inefficient growth (Pirt, 1965). Maintenance energy becomes especially important when growth rates are low. Using the Pirt equation and values for mixed rumen bacteria in chemostats (Isaacson et al., 1975), Russell (2007a) calculated that maintenance energy would account for only 10% of total glucose consumption at the relatively high growth rate of 0.2 h^−1^. However, it would account for 31% total glucose consumption at the low growth rate of 0.05 h^−1^.

Because bacteria pass with digesta, their growth rate increases with increasing digesta passage rate in the rumen. Increasing passage rate by nutritional manipulation would be one strategy to decrease the relative impact of maintenance energy and improve growth efficiency. This is a facile strategy, however, because increasing passage rate, such as by grinding forage, decreases feed digestibility (Van Soest, 1994). A more defensible strategy to improving growth efficiency is to target other non-growth functions, such as energy spilling and reserve carbohydrate synthesis.

### Occurrence of energy spilling in pure cultures

Although maintenance functions depress microbial growth efficiency, only energy spilling can explain very low efficiency during carbohydrate excess (and other growth-limiting conditions). Russell (1986) demonstrated energy spilling by pulse-dosing rumen bacterial cultures with glucose. Cultures fermented excess glucose rapidly, produced very little protein (growth efficiency approached 0), and dissipated (spilled) energy by producing heat. Van Kessel and Russell (1996) reported that mixed rumen bacteria fermented glucose 10-fold faster when spilling energy, implying that spilling could be a significant sink for ATP.

Spilling occurs in organisms across all three domains of life (Table 2). Spilling has been demonstrated extensively in a few rumen (*Streptococcus bovis*) and non-rumen (*Escherichia coli, Klebsiella aerogenes*) bacteria. Though evidence is less extensive, it also appears to occur in protozoa, fungi, and methanogens, suggesting broad importance. In almost all cases, single-species cultures, not mixed communities, have been studied.

**Table 2:** **Occurrence of energy spilling in microbes^a^**.

**Species**	**Ruminal isolate**	**Heat production or growth yield evidence**	**Mechanism (type of cycling)**	**Notes**	**References**
**BACTERIA**					
*Escherichia coli*	N	Lower growth yield per ATP under Mg, P, S, K vs. C-limitation	NH_3_/NH^+^_4_/H^+^	Mechanism applies during high NH_3_/NH_4_ and low K^+^	Buurman et al., 1991
	N	Lower growth yield per ATP of wild-type vs. K^+^ transport mutant	K^+^	Mechanism applies during low K^+^	Mulder et al., 1986
	N	ND	NH_3_/NH^+^_4_/H^+^/K^+^	Mechanism applies during low NH_3_/NH^+^_4_	Russell and Cook, 1995
*Klebsiella aerogenes*	N	Lower growth yield per ATP under NH_3_, P, S, or K vs. C-limitation	ND		Neijssel and Tempest, 1976; Teixeira de Mattos and Tempest, 1983
*Fibrobacter intestinalis*	N	ND	Glycogen		Matheron et al., 1998
*Fibrobacter succinogenes*	Y	ND	Glycogen		Gaudet et al., 1992; Matheron et al., 1998
*Prevotella bryantii*	Y	Heat production rose rapidly during glucose excess	ND	Growth and reserve carbohydrate not accounted explicitly	Russell, 1986
*Selemonas ruminantium*	Y	Heat production rose rapidly during glucose excess	ND	Growth and reserve carbohydrate not accounted explicitly	Russell, 1986
*Streptococus bovis*	Y	Heat production not accounted by maintenance energy or growth	H^+^		Russell and Strobel, 1990; Cook and Russell, 1994; Bond and Russell, 1996, 1998
Mixed rumen bacteria	Y	Lower growth yield per hexose under NH_3_-N vs. amino-N	Not defined	Reserve carbohydrate or other cell composition changes not accounted explicitly	Van Kessel and Russell, 1996
**PROTOZOA**					
*Isotricha protostoma*	Y	ND	Glycogen		Prins and Van Hoven, 1977
*Dasytricha ruminantium*	Y	ND	Glycogen		Van Hoven and Prins, 1977
**FUNGI**					
*Saccharomyces cerevisiae*	N	Lower growth yield per ATP under glucose excess	ND		Van Urk et al., 1988
	N	Higher heat production and lower growth yield under N vs. glucose-limitation	ND	Reserve carbohydrate not accounted explicitly	Larsson et al., 1993
	N	ND	Trehalose	Mechanism applies under heat shock	Hottiger et al., 1987
**ARCHAEA**					
*Methanobacterium thermoautotrophicum*	N	Lower growth yield per CH_4_ under H_2_- or CO_2_- excess vs. limitation	ND	Reserve carbohydrate or other cell composition changes not accounted explicitly	Schönheit et al., 1980; Morgan et al., 1997
**MIXED OR UNDEFINED**					
Mixed rumen microbes	Y	Heat production not accounted by endogenous metabolism or reserve carbohydrate	ND	Endogenous metabolism used as proxy for maintenance energy	Hackmann et al., 2013a

a *N, no; Y, yes; ND, Not determined*.

Spilling can be evidenced by depressed growth efficiency or elevated heat production in response to excess carbohydrate (Table 2). Carbohydrate excesses have been generated by pulse dosing glucose or growing cells under limitation of an anabolic substrate (e.g., N, Mg, P, S, K). Spilling can also be evidenced in response to (1) ammonia-N replacing amino-N and (2) excess H_2_ or CO_2_ (for methanogens). When measuring changes in growth efficiency and heat production, one must account for any changes in maintenance energy, reserve carbohydrate, and growth (cf. Figure 1), though this is sometimes not done (Table 2).

The mechanism of spilling is by futile cycles of ions, glycogen, or trehalose (Table 2). The best-elucidated mechanism is for *S. bovis*, for which spilling occurs by futile cycling of protons. This cycling results from growth limitation and a cascade of biochemical events (Russell, 2002). Specifically, a growth limitation decreases use of ATP for protein synthesis, increasing fructose-1,6-bisphosphate, decreasing intracellular phosphate, increasing the absolute value of Gibbs energy of ATP hydrolysis, increasing activity of a proton-pumping ATPase, and decreasing membrane resistance to protons. The net result is heat, with no work done by the protons.

For *E. coli*, the mechanism of energy spilling is by cycling of K^+^ or NH^+^_4_ or a combination thereof, depending on the extracellular concentration of K^+^ and NH^+^_4_ (Table 2). For many other organisms, cycling of glycogen and trehalose may occur (Table 2). Such cycling implies energy spilling can occur, even though spilling often has not been measured directly (growth efficiency and heat production were often not determined; Table 2). Some authors have proposed that fructose-6-phosphate/fructose-1,6-bisphosphate (Otto, 1984) or other substrates (Newsholme et al., 1984) can be cycled. However, such cycling is difficult to establish under physiological conditions (Russell and Cook, 1995; Portais and Delort, 2002).

The biological rationale for spilling is not clear: why expend what can be conserved? Some have proposed spilling allows microbes to rapidly catabolize substrate and dissipate energy in order to (1) deprive competitors of energy or (2) hasten re-initiation of growth when a growth limitation is released (Tempest, 1978; Russell and Cook, 1995). Although all futile cycles dissipate energy and thus cause spilling, this spilling may be an unfortunate byproduct, not the primary function, of some futile cycles. Rather, the function of some futile cycles may be to sensitively control the net flux of metabolites (Newsholme and Crabtree, 1975). Regardless of function, energy spilling and futile cycles are wasteful from the perspective of growth efficiency, and their impact on growth of the rumen microbial community needs to be elucidated.

### Occurrence of energy spilling in mixed cultures

Many pure cultures have been demonstrated to spill energy, but until recently, examples of mixed communities that spill energy were few (Table 2). In earlier studies, Van Kessel and Russell (1996) suggested that rumen bacteria spilled more energy when grown under ammonia-N vs. amino-N limitation. Their approach assumes constant cell composition, and they did not measure reserve carbohydrate. Some energy may have in fact been directed to reserve carbohydrate synthesis, not spilling. Chen et al. (2000) induced spilling in activated sludge by adding a protonophore, but spilling was not measured under more physiological conditions.

**Figure 2:**
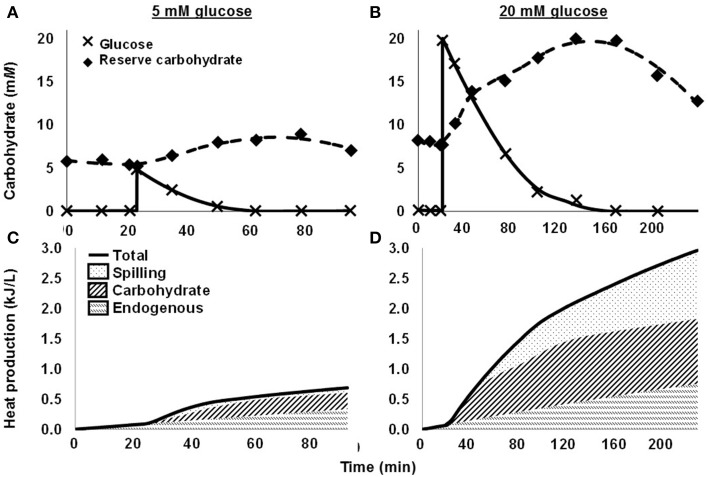
**Energy spilling and other responses of mixed rumen microbes to pulse dose of glucose. (A,C)** 5 m*M* glucose. **(B,D)** 20 m*M* glucose. **(A,B)** Glucose in media and reserve carbohydrate. Glucose was dosed at 20 min. **(C,D)** Heat production, including heat accounted by endogenous metabolism, synthesis of reserve carbohydrate, and energy spilling. Data are for 1 cow, and each glucose concentration represents a single experiment. Figure adapted from Hackmann et al. (2013a).

More recently, we quantified spilling for mixed rumen microbes (Hackmann et al., 2013a). When we washed cells with N-free buffer and dosed them with glucose, they consumed glucose rapidly, accumulated reserve carbohydrate, and did not grow (protein remained constant) (Figures 2A,B). When we dosed a moderate concentration of glucose (5 m*M* glucose), no spilling was detected, as nearly all heat production (93.7%) was accounted by reserve carbohydrate synthesis and endogenous metabolism (a proxy for maintenance energy) (Figure 2C). When we dosed a high concentration of glucose (20 m*M*), energy spilling was not detected immediately, but it accounted for a significant amount of heat production approximately 30 min after dosing (Figure 2D). Energy spilling accounted for as much as 38.7% of heat production in one incubation.

### Identity and occurrence of reserve carbohydrate

Rumen microbes can accumulate prodigious amounts of reserve carbohydrate. It can exceed 50% of cell weight for pure cultures of both rumen (Russell, 1998) and non-rumen (Preiss and Romeo, 1989) bacteria. Some protozoa (Isotrichidae) accumulate enough reserve carbohydrate to turn opaque (Williams and Coleman, 1992). Net accumulation of glucose into reserve carbohydrate can exceed 50% for mixed microbes (Hackmann et al., 2013a) and protozoa (Denton et al., 2015). The identity of this reserve carbohydrate is glycogen [glucan with (α1→4) and (α1→6) linkages] and appears ubiquitous across rumen bacteria, fungi, and protozoa (Table S1). Synthesis of these prodigious amounts of reserve carbohydrate irreversibly expends ATP, decreasing ATP available for protein synthesis (Figure 1). This lowers growth efficiency on a protein basis (g protein/mmol ATP), as it probably does on a dry matter basis (g DM/mmol ATP) (explained later).

### Dynamics of accumulation

In the rumen, reserve carbohydrate accumulates immediately after feeding (during carbohydrate excess), and then is mobilized thereafter (during carbohydrate limitation). This is observed for both rumen bacteria (Figure S2) and protozoa (Figure S3) (Jouany and Thiven, 1972; McAllan and Smith, 1974; Williams and Harfoot, 1976; Leedle et al., 1982). These dynamics are most dramatic for high-grain and low-N diets, which create large carbohydrate excesses after feeding (cf. basal diet + urea vs. basal diet in Figure S2).

For *in vitro* batch culture (Figures 2A,B), where conditions can be better defined, we observed that rumen microbes showed similar dynamics of accumulation and mobilization as *in vivo* (Figures S2, S3). When we washed mixed rumen microbes with N-free buffer and dosed glucose, reserve carbohydrate immediately accumulated (Figures 2A,B). At peak accumulation, microbes had incorporated 59.5% of glucose carbon in reserve carbohydrate when we dosed 5 m*M* glucose. The value was lower (52.6%) for 20 m*M* glucose. After glucose was exhausted, reserve carbohydrate quickly declined (Figures 2A,B).

**Figure 3:**
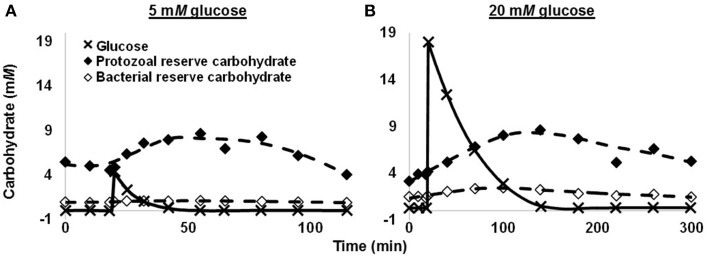
**Glucose use and reserve carbohydrate accumulation of a mixture of rumen protozoa and bacteria in batch culture. (A)** 5 m*M* glucose. **(B)** 20 m*M* glucose. Data are for 1 cow, and each glucose concentration represents a single experiment. Figure adapted from Denton et al. (2015).

In a subsequent batch culture study, we observed that protozoa, not bacteria, were responsible for most glycogen accumulation. In this study, we performed competition experiments in which mixtures of protozoa and bacteria were first dosed with glucose, and then at intervals the two groups were separated for glycogen analysis. When the mixtures were dosed with a moderate concentration of glucose (c. 5 m*M*), protozoa incorporated 58.7% of glucose carbon in reserve carbohydrate at time of peak reserve carbohydrate (Figure 3A). Bacteria had incorporated only 1.7%. When we dosed mixtures with a high concentration glucose (20 m*M*), the amounts incorporated were 21.4% for protozoa and 5.0% for bacteria, respectively (Figure 3B). Protozoa would thus appear the predominant group accumulating reserve carbohydrate. In sum, rumen microbes display a high capacity for accumulating and mobilizing reserve carbohydrate. As explained later, this can expend ATP and lower growth efficiency.

### Relation to growth efficiency

At first consideration, synthesis of reserve carbohydrate should not appear to depress growth efficiency; rather, it would seem to improve it. Glycogen, a common reserve carbohydrate, requires fewer ATP for synthesis than all other cellular macromolecules except lipid (Table 3). Simple arithmetic would suggest more dry matter could be formed when glycogen vs. most other macromolecules are synthesized—i.e., efficiency of growth on a dry matter basis (g DM/mmol ATP) would be higher.

**Table 3:** **ATP required for synthesis of cellular macromolecules**.

**Macromolecule**	**ATP requirement (mmol g^−1^)**
Protein	36.5
RNA	14.6
DNA	18.0
Lipid	1.5
Polysaccharide (glycogen)	12.4

*Calculated from Stouthamer (1973) for growth with glucose, amino acids, and nucleic acid bases, excluding ATP for mRNA turnover and solute transport*.

Simple arithmetic does not consider that reserve carbohydrate accumulation is dynamic. As mentioned, rumen microbes vacillate between (1) reserve carbohydrate synthesis during carbohydrate excess and (2) degradation during carbohydrate limitation. The price paid for such vacillation is expenditure of ATP. For sequential synthesis and degradation of glycogen, 1 net ATP equivalent is expended per glucose (Figure S4). Synthesis of glycogen may cost few ATP compared to most other macromolecules (Table 3), but this low initial cost may be quickly outweighed by this sequential synthesis and degradation. Reserve carbohydrate synthesis could thus lower growth efficiency on a dry matter basis.

Reserve carbohydrate may have been historically overlooked in growth efficiency measurements because most experiments employ chemostats under steady-state conditions (see references in Russell and Cook, 1995). By design, the steady state input of substrate will prohibit vacillation between glycogen synthesis and degradation. Some experiments employ batch cultures (Russell and Cook, 1995), but they are usually terminated during exponential growth, before reserve carbohydrate degradation typically occurs.

Even when reserve carbohydrate would not lower growth efficiency on a DM basis (g DM/mmol ATP), it would always lower it on an N or protein basis (g N or protein/mmol ATP). No matter its course, reserve carbohydrate synthesis (2 ATP/glucose; Figure S4) would reduce ATP available for synthesis of protein and other N-containing macromolecules. This point is indirectly supported by the batch culture experiments of Hall (2011), in which destroying isotrichid protozoa by blending (1) decreased reserve carbohydrate accumulation by 43% and (2) increased growth efficiency on an N basis (g N/g soluble carbohydrate fermented) by 17%. In ruminant nutrition, growth efficiency is usually expressed in terms of protein or N, reflecting that microbial protein accounts for the majority of AA reaching the animal small intestine (Storm et al., 1983).

Although chemostats are more consistent with *in vivo* conditions than batch cultures because the former permit changes in dilution rate, such changes are simultaneous with increasing substrate supply. In contrast, increasing passage rate from the rumen should increase microbial growth rate, at least in part, independently from substrate supply (Dijkstra et al., 1998). As described in that report, increasing growth rate (decreasing division time) is projected to increase growth efficiency (g bacterial DM/g carbohydrate) and decrease the proportion of ATP used to support non-growth functions. Such models would be more accurate with better understanding of how much more reserve carbohydrate is accumulated under different dietary conditions.

### Glycogen cycling

On the surface, rumen microbes would seem to simply (1) synthesize reserve carbohydrate during carbohydrate excess and (2) degrade it during carbohydrate limitation. However, many rumen and non-rumen microbes have been shown to simultaneously synthesize and degrade (cycle) glycogen (Table 2) (Portais and Delort, 2002). This cycling expends ATP, just as sequential synthesis and degradation of glycogen do (Figure S4). It would thus depress growth efficiency.

Although we have discussed reserve carbohydrate synthesis and energy spilling independently, glycogen cycling would link these two functions because it is a form of energy spilling. Glycogen cycling has not yet been demonstrated for mixed rumen communities (Table 2), but we have speculated that it is the mechanism of spilling observed for mixed rumen microbes (Hackmann et al., 2013a).

### Occurrence of carbohydrate excess in the rumen

Both energy spilling and reserve carbohydrate synthesis occur primarily under carbohydrate excess. Carbohydrate is in greatest excess in the rumen for animals fed grain, particularly those transitioning to a high-grain diet, and also for animals fed high-sugar diets. For animals transitioning to a high grain diet, glucose can reach high concentrations [c. 5 m*M* (Ryan, 1964; Mackie et al., 1978)]. Even higher glucose concentrations (18 m*M*) have been reported for animals fed dextrose (Piwonka et al., 1994), and soluble sugar concentrations as high as 69 m*M* have been reported for animals fed beet pulp (Clapperton and Czerkawski, 1969). Concentrations in microenvironments (e.g., around starch granules) may also be high (Kajikawa et al., 1997).

For grain-fed animals, N availability can be low (NRC, 2000), also, and intensify carbohydrate excess (NRC, 2000). Availability of N can also be low for dairy rations with corn silage as the sole source of forage (Vandehaar, 2005). For animals in which N is chiefly in the form of ammonia, carbohydrate excess could be further intensified (Van Kessel and Russell, 1996) because rumen microbes grow far slower with ammonia-N than amino-N (Argyle and Baldwin, 1989; Van Kessel and Russell, 1996). Energy spilling and reserve carbohydrate synthesis would likely depress growth efficiency under these conditions, with spilling being more important at large carbohydrate excesses and reserve carbohydrate more important for smaller excesses (Hackmann et al., 2013a). Spilling has previously been suggested to account for low growth efficiency for high-concentrate diets (Clark et al., 1992).

For animals fed high-forage diets or adapted to grain, carbohydrate excess is relatively small, and glucose concentrations rarely exceed c. 2.5 m*M* (Kajikawa et al., 1997; Saleem et al., 2012). Energy spilling may play a minor role under these conditions, given that we did not detect spilling in batch cultures with glucose concentrations of 5 m*M* (Hackmann et al., 2013a). Reserve carbohydrate accumulation may still be important and decrease growth efficiency, however, given that reserve carbohydrate can still be detected even when cattle are provided low-quality grass diets (Van Kessel and Russell, 1997).

### Other factors depressing growth efficiency

#### Other responses to excess carbohydrate

Rumen microbes may respond to excess carbohydrate in ways other than spilling energy and synthesizing reserve carbohydrate. These other responses include reducing ATP yield by releasing metabolic intermediates (overflow metabolites) and shifting to catabolic pathways that yield less ATP (Russell, 1998). Responses are similar for non-rumen microbes (Tempest and Neijssel, 1984; Preiss and Romeo, 1989; Russell and Cook, 1995; Russell, 2007b).

#### Recycling of microbial protein

Recycling of microbial protein is another factor that depresses growth efficiency. As much as 50% of microbial protein is degraded to non-protein nitrogen in the rumen and recycled (Wells and Russell, 1996; Oldick et al., 2000). Protozoal predation, autolysis, and bacteriophages may all be causes (Wells and Russell, 1996). Most recycling has been thought to be mediated by protozoa predation, based on lysis of pure bacterial cultures in presence and absence of rumen fluid with protozoa (Wallace and McPherson, 1987). However, removing protozoa from the rumen was subsequently shown to have no effect on bacterial N recycling *in vivo* (Koenig et al., 2000). Firkins et al. (2007) reasoned that protozoa-mediated recycling of microbial protein is lessened with increasing passage rates by high-producing animals compared with some of the studies with low intakes or predictions based on measurements *in vitro*.

#### Exopolysaccharide synthesis

In addition to synthesizing reserve carbohydrate, rumen bacteria can synthesize exopolysaccharides (Hobson and Macpherson, 1953, 1954; Costerton et al., 1974). One exopolysaccharide, dextran, is synthesized by *S. bovis* when given excess sucrose (Bailey and Oxford, 1958; Cheng et al., 1976), and it forms part of the “slime” observed in grain-fed animals with frothy bloat (Cheng et al., 1976). Up 80% of glucose in sucrose can be directed into its synthesis, but synthesis does not require ATP (Bailey, 1959; van Hijum et al., 2006), and there is no ATP cost of transport because synthesis occurs extracellularly (van Hijum et al., 2006). Thus, dextran formation would not depress growth efficiency on an ATP basis. *Ruminococcus albus* also forms an exopolysaccharide, but synthesis of this exopolysaccharide has been estimated to account for <4% of ATP of total used for synthesizing cell components (Weimer et al., 2006). Many other rumen bacteria form exopolysaccharides (Hobson and Macpherson, 1953, 1954; Costerton et al., 1974), but their formation has not been quantified, and their impact on growth efficiency remains unknown.

#### Cellodextrin efflux

At least some cellulolytic bacteria expend ATP on cellodextrin efflux, which should depress their growth efficiency. The cellulolytic *Fibrobacter* spp. synthesize cellodextrins intracellularly, but these can be lost by extracellular efflux (Wells et al., 1995). The cell expends ~4/3 ATP from combined costs of synthesis (1 ATP) and active transport (~1/3 net ATP) (Figure S5). This ATP expended on cellodextrin synthesis would be recovered by non-cellulolytic bacteria after they take up the cellodextrin (assuming that transport is the exact opposite of efflux). Consequently, cellodextrin efflux should depress growth efficiency of some cellulolytics, not the microbial population as a whole.

Efflux of maltodextrins, not only cellodextrins, has been observed for *F*. *succinogenes* (Matulova et al., 2001; Nouaille et al., 2005). Maltodextrin efflux would be expected to expend ATP just as does cellodextrin efflux, but the exact expenditure is unknown because the pathway for maltodextrin synthesis is uncertain (cf. Matulova et al., 2001). Consequently, maltodextrin efflux likely depresses growth efficiency of some cellulolytics, but its exact impact on the cellulolytics and microbial population as a whole remains unknown.

Cross-feeding of cellodextrins is often depicted as being beneficial to the non-cellulolytics but also the cellulolytic populations by removing end-product inhibition of cellobiose on cellulases (Russell et al., 2009). As documented by those authors' model, increasing cellulolysis also diverts an increasing proportion of carbon toward cell growth and away from fermentation. However, if growth of the community is uncoupled by limitations of nitrogen or other growth factors, then an increasing proportion of carbon should be directed away from cell growth and toward SCFA, promoting energy spilling. In most studies measuring energy spilling, the medium was buffered. If total SCFA production was fast enough to decrease the ruminal pH below approximately 6.0, as can happen in the rumen, the proton gradient across the cell membrane could inhibit cellobiose transport by cellulolytics and thereby inhibit fiber degradation (Russell et al., 2009).

#### Cellobiose hydrolysis or transport

Russell (2002) described various mono or disaccharide transport mechanisms, including the phosphoenolpyruvate:phosphotransferase systems (PEP-PTS). Active transport also increases the ATP cost, but active transport of a disaccharide can have a decreased ATP charge if it is transported prior to hydrolysis into monosaccharides, and the ATP charge can be further decreased if disaccharide transport is coupled with a phosphorylase. Whether a phosphorylase or a PEP-phosphotransport system phosphorylates the sugar, this ATP charge is recovered by negating ATP required for a hexokinase reaction. Not all pure cultures were shown to express PEP-PTS transport of sugars (Martin, 1994). In mixed ruminal microbes, though, glucose or other sugars were assumed to be transported primarily by the PEP-PTS system (Kajikawa et al., 1997). Glucose is typically not fed to ruminants, but primarily is the product of cellulose and starch degradation. Some cellobiose was transported into mixed bacterial cells by a PEP-PTS but also by ATP-expending transporters (Kajikawa and Masaki, 1999). Increasing availability of maltose or maltodextrins for transport might lead to increased SCFA production and lower ruminal pH. Cellulolytic bacteria can be inhibited by pH < 6.0 through depressed adherence to cellulose, decreased cellobiose transport, or from inhibitory membrane gradients of anions or protons (Russell et al., 2009).

Genomics-based analyses have revealed a much more complicated mechanism in which genes are expressed as polysaccharide utilization loci (Wang et al., 2013). Di- or oligosaccharides from hydrolyzed cellulose can be transported in gram-negative *F. succinogenes* (Suen et al., 2011), gram-positive *Ruminococcus flavefaciens* (Flint et al., 2008), and from hydrolyzed hemicellulose in gram-positive *Butyrivibrio proteoclasticus* (Dunne et al., 2012), respectively, likely from a combination of ABC-transporters and those linked to phosphorolytic cleavage. Substrate source and availability probably regulates expression of many of these transporters (Bond et al., 2012). Based on metagenomics screening of cellulases and xylanases, many genes were novel, but a relatively high proportion were reputed transporters (Wang et al., 2013). There is likely periplasmic sequestration of oligosaccharides from cellulose (White et al., 2014), hemicellulose (Morgavi et al., 2013), and starch (Rosewarne et al., 2014). Therefore, the net ATP cost of di- and monosaccharide transport into cytosol is not fully known but probably varies with substrate availability.

Rapid growth decreased glycogen concentration of *P. bryantii* B_1_4 (formerly *P. ruminicola*) (Lou et al., 1997b). However, with slower growth, maltose increased glycogen concentration more than when using glucose as substrate. When using maltose as substrate, maltose phosphorylase activity (which couples transport with phosphorylation of a glucose moiety) was increased and glycogen accumulated even when N was not limiting and growth rates increased. Similar results were detected when grown on cellobiose. In another study, growing *P. bryantii* on maltose or cellobiose increased the activity of UDP-glucose pyrophosphorylase and glycogen synthase compared with growth on sucrose or glucose (Lou et al., 1997a). Thus, transport and metabolism of maltose to glucose-1-phosphate was associated with glucose-1-phosphate polymerization into glycogen. Although poorly studied with mixed microbes, either gene expression of the reversible enzyme phosphoglucomutase or an accumulation of glucose-6-phosphate could help push synthesis of glycogen. UDP-glucose pyrophosphorylase (enzyme prior to glycogen synthase) was activated by fructose-1,6-phosphate in *P. bryantii* (Lou et al., 1997a). Pulse doses of glucose decreased metabolism of cellobiose and activity of cellobiose phosphorylase in *P. bryantii*, and vice versa (Lou et al., 1996). Thus, accumulation of disaccharides from rapid hydrolysis of cellulose or starch could stimulate glycogen synthesis in ruminal bacteria and thereby increase glycogen cycling as a means of energy spilling. *Prevotella bryantii* is well-known for energy spilling among cultivated prevotellas (Russell, 1992), although uncharacterized prevotellas often predominate in the rumen (Firkins and Yu, 2015).

#### Branched short-chain fatty acids

The primary cellulolytics, most of which were characterized decades ago, have various requirements for growth factors such as branched chain SCFA and phenyl-substituted SCFA that are provided by secondary colonizers, which generally are much more proteolytic (Stewart et al., 1997). Despite the importance of branched chain SCFA required by isolates of cellulolytics, feeding these compounds *in vivo* primarily was associated with post-absorptive rather than ruminal responses (Andries et al., 1987). Given the importance of primary (or “keystone”) colonizers (Ze et al., 2013), such a lack of ruminal response can be reconciled with a broader view of how bacteria are stimulated by preformed AA to better balance anabolic and catabolic pathways during growth (Russell and Cook, 1995).

#### Peptides and amino acids vs. ammonia-N

Although there seems to be little difference between AA and peptides for mixed cultures, many pure cultures of bacteria are stimulated by provision of small peptides rather than free AA (Wallace et al., 1997). For example, *R. albus* was shown to transport peptides but not AA (Kim et al., 2014). Peptides had a minor effect on this strain's growth rate, which was maximized at about 0.9/h. In contrast, *S. bovis*, which is known for rapid growth on starch or sugar, had growth rate of 0.9/h with NH_3_ that was stimulated to 1.6/h when AA were provided (Russell, 1993). In that study, incremental growth was synchronized with incremental boluses of glucose compared with a single dose, and the maximal growth available was particularly limited when glucose doses were incrementally staggered and when ammonia replaced AA. In contrast with pure cultures, when AA were provided with a mixture of carbohydrates as substrate, growth of mixed ruminal microbes was still stimulated by approximately 50% compared with providing NH_3_ even when the growth rates of the mixed cultures were lower (i.e., from 0.25–0.30 increased to 0.40–0.45/h) (Kajikawa et al., 2002). Because the latter stimulation is from growth rates below the threshold above which AA were expected to stimulate growth (Van Kessel and Russell, 1996), even growth of cellulolytics limited by rate of cellulolysis can be stimulated. In contrast with prior expectations, preformed AA now are considered as potentially stimulatory for consortia of microbes degrading fiber (Newbold, 1999).

#### Supply and profile of preformed amino acids

Although energy is required for bacteria to synthesize AA, this energy cost is small; when preformed AA are limiting, though, growth rate is slowed, and the balance of anabolic and catabolic rates leads to increased energy spilling (Russell and Cook, 1995). As depicted in Figure S6 (only for NH_3_ assimilation, not for transamination of other AA), Ala, Glu, and Gln are the primary AA formed from assimilation of ammonia in the rumen. Relative fluxes of these amination reactions depend on the Michaelis constant (*K_m_*) of ammonia for those enzymes but also based on transcription of ammonia-assimilating enzymes (Morrison and Mackie, 1997). Kim et al. (2014) documented an excellent example of transcriptional control of ammonia-assimilating enzymes in *R. albus*. Although bacteria can make most of their AA, gelatin (which has a poor profile of Leu and the aromatic AA) decreased growth rates of mixed bacteria (Van Kessel and Russell, 1996) and increased energy spilling.

Although AA are stimulatory to growth (Kajikawa et al., 2002), an imbalance of branched or aromatic AA was worse than deletion of the entire group of AA. Bacteria can partially control the flux of AA biosynthetic pathways (Figure S6), but congruent pathways likely antagonize availability of closely related AA when they are out of balance. Moreover, pathways for biosynthesis of AA intersect with central metabolic pathways used in fermentation. Most rumen bacteria lack a complete TCA cycle and must use what would be considered both forward and backward reactions of that cycle (if it was complete) to form α-ketoglutarate (Wallace et al., 1997). Thus, the alternating directional flux of this interrupted cycle must be able to provide the mix of intermediates for anabolism while intersecting with catabolic reactions to make ATP to drive anabolism. Because many of these intermediates are produced through dehydrogenases, the NADH/NAD ratio must be resolved with catabolic fermentation reactions. In support, the NADH/NAD ratio can regulate the deamination of reduced AA, in particular the branched chain AA, as this deamination produces NADH (Hino and Russell, 1985).

Part of the difficulty in assessing how AA profile affects growth efficiency lies in how various studies were done. For example, bolus doses of isotopically labeled AA or peptides mostly yielded catabolism for energy, except for Leu, Tyr, and Phe (Armstead and Ling, 1993; Atasoglu et al., 1999). Only deletion of Leu (not deletion of other AA) decreased bacterial growth (Atasoglu et al., 2003), but Atasoglu et al. (2004) noted that Ile, Phe, Lys, and (to a somewhat lesser effect) Leu were incorporated into bacterial protein to a greater extent than were other AA. These results are consistent in that the branched chain and aromatic AA have similar metabolism within their respective groups and are more limiting than other AA.

In some of these types of studies, the peptide or AA were dosed as both the primary nitrogen and carbon source. Most of the pure cultures of predominant saccharolytic bacteria with more moderate deaminative activity did not grow well when peptides or AA provided the sole substrate (Wallace et al., 1997). Those authors reasoned that proteolysis by saccharolytic bacteria might be more important in exposing carbohydrate. Compared with other studies, there was relatively high assimilation of preformed AA in the study of Atasoglu et al. (2004) because they dosed labeled AA concomitantly with carbohydrate substrate. Those authors also noted how preformed AA, after being taken up by cells, can be catabolized if not assimilated. Firkins et al. (2015) postulated that Met is incorporated into cellular protein but that surplus intracellular Met recycles intracellularly and likely exchanges with an extracellular pool. Secretion or leakage of AA is likely when AA are high relative to concentrations needed for protein synthesis or AA are imbalanced.

Some discrepancies among studies evaluating amount or profile of preformed peptides or AA for bacteria also might be a result of inadequate adjustment time upon removal of protozoa. Protozoa appear to contain high deaminase activity (Wallace et al., 1997). However, protozoa might excrete up to half of the degradation fragments from the bacterial protein it consumes (Hristov and Jouany, 2005). Little is known about the AA profile of excreted bacterial proteins (rich in cell wall proteins?), and the excreted peptides might also be mixed with excreted but active protozoal peptidases. Although clearly important, predation of bacteria and lysis rates of protozoa probably have been exacerbated under the *in vitro* conditions in which normal substrate was replaced with various bacterial strains to quantify bacterial predation by loss of planktonic bacterial counts (Diaz et al., 2014). The hyperammonia-producing bacteria make a significant contribution to the total deamination activity in the rumen but are in low numbers (Walker et al., 2005). Thus, effects on growth rate of this group would be masked in mixed cultures.

In studies that have separated protozoal and bacterial fractions, there often is synergistic action when these two groups are added together (Walker et al., 2005). Defaunation (removal of protozoa) consistently decreases ruminal ammonia concentration compared with faunated controls, with explanations typically assuming exclusion of protozoal proteolytic and deaminative enzymes (Hristov and Jouany, 2005). However, an alternative explanation is that defaunation should increase the abundance of bacteria most of which assimilate ammonia, whereas protozoa do not. Ruminal protozoa can limit efficiency of microbial protein synthesis in the rumen through predation of bacteria, but there is a large gap in studies with defaunated animals at production-level intakes (Firkins et al., 2006). Recent improvements in methods allow the extraction of metabolically active protozoa with minimal bacterial contamination (Denton et al., 2015), which is critical because protozoa can degrade endogenous protein (Forsberg et al., 1984).

#### Asynchrony and primary and secondary colonizers

The rumen microbiome has received considerable attention to optimize fiber degradation and minimize problems with ruminal acidosis (Firkins and Yu, 2015). In contrast with many studies evaluating the synchrony of nutrients for pure cultures or simple communities, the rumen is far more complex. To maintain a balanced consortium, asynchrony of carbohydrate and nitrogen sources can have a profound rippling effect through entire communities of ruminal microbes.

Physical and environmental limitations alter the degradation and usage of carbohydrate by microbes in the rumen. Degradation rates of crystalline cellulose (Weimer, 1996) by cellulases are probably limited by surface area rather than enzymatic capacity (Fields et al., 2000) and therefore are much slower than the potential growth rates by cellulolytics on the resultant cellobiose or cellodextrins (Shi and Weimer, 1997). Secondary degraders cross-feed from the degradation products produced by primary degraders of starch (Cotta, 1992), cellulose (Russell, 1985), and hemicellulose (Cotta and Whitehead, 1998). Excessive degradation of carbohydrate can decrease cellulose degradation by primary degraders; it can do so by decreasing pH or leading to depletion of growth factors (Weimer, 1996; Mouriño et al., 2001).

## Role of short-chain fatty acid interconversions in mixed ruminal communities

The carbon used for substrate should be reconciled with carbon recovered as products, including SCFA and cellular growth. Many SCFA interconversions and usage for anabolism allow anaerobic bacteria to fill intermediates of metabolites. Exchanges of SCFA, especially between acetate and butyrate, are to be expected (Firkins et al., 2006). Therefore, researchers have used radio or stable isotopes to support the integration of anabolic and catabolic fluxes. Indeed, an estimated 28% of [2-^13^C]acetate infused into cattle was not recovered as absorbed acetate (Kristensen, 2001). Many of these exchanges allow microbes to reoxidize reducing equivalents and have little net effect on growth of the community. For human fecal bacteria, numerous interconversions are possible but with a major conversion of acetate to butyrate (Falony et al., 2006; Morrison et al., 2006). Unfortunately, there is much less known for ruminal bacteria.

### Cycling of acetate during butyrate production in butyrivibrios

Exogenously derived acetate can be used in a cycle to produce butyrate from acetyl coA (Diez-Gonzalez et al., 1999). In that cycle (Figure S7A), exogenous acetate would not directly wind up in butyrate but would aid in reactions transferring coenzyme A. The butyrivibrios, which are the main characterized bacteria involved in biohydrogenation, cluster taxonomically by either high or low expression of butyrate kinase (Paillard et al., 2007). The cluster with high butyrate kinase activity (cf. Figure S7B) comprised the only stearate producers so far described, whereas the cluster with the lower butyrate kinase activity (cf. Figure S7A) could not complete biohydrogenation to stearate. That latter group expressed more butyryl coA-acetyl coA transferase (butyryl coA + acetate → butyrate + acetyl coA). Presumably, the acetyl coA would then produce acetyl-phosphate and then generate ATP as acetyl kinase yields acetate (Diez-Gonzalez et al., 1999), with the acetate then becoming available for another cycle. The two groups also can be characterized based on pyruvate flux. The group expressing butyryl kinase produces little lactate and produces more acetate than the other group. In contrast, the group expressing the butyryl coA-acetyl coA transferase enzyme increases lactate production with increasing concentration of fructose-1,6-phosphate (rapid glycolysis) but takes up considerable acetate. The increasing acetate uptake would indicate that some acetyl coA must be replenishing acetyl coA pools for butyrate production and perhaps other anabolic reactions such as fatty acid biosynthesis.

Although the butyrivibro group expressing the coA transferase was only inhibited by higher concentrations of linoleic acid, when one representative isolate was dosed with linoleic acid above the inhibition threshold, the various acyl coA pools and ATP production dramatically decreased (Maia et al., 2010). Based on that response, those authors proposed a metabolic inhibition rather than disrupted membrane function to explain the toxicity from linoleic acid. This cluster of butyrivibrios produced more lactate in pure cultures, and lactate exacerbated inhibition by linoleic acid (Paillard et al., 2007). A disrupted cycle involving acetate uptake and the butyryl coA-acetyl coA transferase reaction could help explain the depleted acetyl coA pools in the study of Maia et al. (2010).

Increased starch fermentability is well-known to shift biohydrogenation away from the trans-11 pathway used by most butyrivibrios and toward the trans-10 18:1 pathway used by as yet poorly characterized bacteria (Jenkins et al., 2008). To our knowledge, energy spilling has not received much attention with butyrivibrios. We note the commonality for fructose-1,6-bisphosphate accumulation to activate lactate dehydrogenase in both the lactate-producing butyrivibrios (Diez-Gonzalez et al., 1999) and for this stimulation of lactate production to coincide with energy spilling through proton cycling in *S. bovis* (Bond and Russell, 1998). Human gut bacteria closely related to *Butyrivibrio fibrisolvens* were projected to produce butyrate through a scheme projecting a proton motive force for subsequent ATP synthesis (Louis and Flint, 2009). A research question to be tested is whether or not the butyrivibrios (especially the stearate producers) might be inhibited by higher starch fermentability because of their inability to spill energy. In contrast, might the likely candidates to biohydrogenate linoleic acid through the trans-10 pathway (i.e., *Propionibacterium, Streptococcus*, and *Lactobacillus*; (Jenkins et al., 2008) be less inhibited by high starch fermentability and more equipped to spill energy.

If bioactive fatty acids accumulate enough to inhibit butyrivibrios, then methanogens also should be affected. Rather than sinking reducing equivalents into biohydrogenation, H_2_ is a more favorable and important sink (Jenkins et al., 2008). To our knowledge, detailed studies with polyunsaturated fatty acids have not been done. However, medium chain fatty acids inhibit methanogens by disrupting ion gradients (Zhou et al., 2013). In that report, despite the bolus doses of these fatty acids, many methanogens were stained still active. Firkins and Yu (2015) described the poor relationship between methane production and abundance of methanogens.

### *De novo* synthesis of fatty acids

Exogenous [2-^13^C]acetate was elongated to butyrate and a variety of longer chain fatty acids in bacterial samples (Kristensen, 2001). Notable recovery was in the odd and anteiso fatty acids needed for bacterial membranes and with minimal isotope recovery in palmitic and stearic acids. Although bacterial long chain fatty acid synthesis was suggested as a mechanism for aerobic bacteria to store excess energy (Bas et al., 2003), the main benefit might be in allowing acetate production to provide ATP in fermentation with subsequent usage of acetate in CoA transferase reactions that consume reducing equivalents while elongating fatty acids during *de novo* synthesis (Duncan et al., 2002). *De novo* fatty acids would clearly be an important sink for carbon diverted from fermentation but also for reducing equivalents derived by fermentation, so hydrogen and carbon recovery models should be reconsidering factors affecting fatty acid biosynthesis vs. fatty acid uptake.

Supplemental fat can improve efficiency of microbial protein synthesis either by inhibition of protozoal predation on bacteria or by alleviation of the need for *de novo* fatty acid synthesis (Hanigan et al., 2013), which would allow more diversion of carbon toward ATP-generating fermentation rather than anabolism. Ruminal bacteria are thought to lack desaturase enzymes and must rely on methylated long chain fatty acids to maintain membrane fluidity (Russell, 2002). As described in that source, branched chain SCFA are elongated in biosynthetic reactions and are therefore important growth factors for many bacteria. In contrast with bacteria, ruminal protozoa can take up more dietary fatty acids and rely less on biosynthesis (Karnati et al., 2009).

### Shifts in fermentation pathways corresponding with growth rate

Fermentation of lactate produced by another microbe is an important cross-feeding mechanism to help buffer against ruminal acidosis (Nagaraja and Titgemeyer, 2007). They explained that, when substrate increases, *S. bovis* shifts fermentation toward lactate to increase ATP yield per time while decreasing ATP yield per glucose fermented. With increasing lactate production, *Selenomonas ruminantium* produces propionate via succinate, and *Megasphaera elsdenii* produces propionate via acrylate; these species are regarded as among the most important of the characterized lactate-utilizing bacteria in the rumen (Nagaraja and Titgemeyer, 2007). Exogenously supplied lactate would be labeled as propionate in different ways, depending on the pathway by those two species (Counotte et al., 1983).

Studies have elucidated regulation of lactate metabolism in some pure cultures of bacteria, and enzymatic pathways likely depend on ATP status. The intracellular ATP concentration or some similar energy gauge apparently regulates lactate metabolism in *S. ruminantium* (Asanuma and Hino, 2001). Higher ATP was proposed to allosterically activate pyruvate kinase to stimulate lactate production (which would decrease ATP yield from glucose). In contrast, phosphoenolpyruvate (PEP) carboxykinase (PEPCK; PEP → OAA) would be induced with lower ATP concentration. Using PEPCK allows GTP synthesis and routes carbon to pyruvate through succinate. With the lactate producer *S. bovis*, high ATP concentration represses pyruvate formate lyase (converting pyruvate to acetate) but induces lactate dehydrogenase to produce lactate (Asanuma and Hino, 2002). Along with allosteric activation of lactate dehydrogenase (Bond and Russell, 1996) and energy spilling (Bond and Russell, 1998), *S. bovis* can greatly increase its rate of lactate production when glucose concentration increases.

Lactate fermentation in the rumen is more complicated because lactate is produced in both D and L stereoisomers (Nagaraja and Titgemeyer, 2007). Glucose concentration can influence the fermentation pathway of these stereoisomers (Weimer and Moen, 2013). In that latter study, a strain of *M. elsdenii* fermented lactate to acetate or propionate, but when the lactate was depleted, the strain fermented glucose to butyrate and valerate. Specifically, the strain fermented glucose to acetyl-CoA, then acetate and propionate were elongated to butyrate and valerate by their condensation with acetyl-CoA. CoA transferases appear to allow metabolic versatility in *M. elsdenii* (Prabhu et al., 2012).

Shifts in metabolism are not unique to lactate producers and consumers. In *R. flavefaciens*, the primary route of OAA formation seems to be via PEPCK, but pyruvate carboxylase activity (i.e., pyruvate → OAA) increased moderately with increasing growth rates, even though OAA derived this way would decrease ATP yield compared with pyruvate through PEPCK (Shi et al., 1997). Pyruvate is the route for acetate production (which yields more ATP than succinate considering ATP production by methanogens). Pyruvate carboxylase could also produce OAA as the precursor for Asp and several other AA (Figure S6). Thus, catabolic fermentation to yield ATP for anabolic reactions might be better balanced with catabolic fermentative routes by fluxing glucose-carbon through pyruvate.

Exogenous carbon dioxide is used and produced in propionate production through succinate (Mountfort and Roberton, 1978). Those authors also detected label from [2-^14^C]acetate in succinate. Shi et al. (1997) projected CO_2_ conversion to formate in a scheme to help *R. flavefaciens* resolve reducing equivalents during different phases of growth as affected by substrate supply.

### Thermodynamic control of interconversions

Ungerfeld and Kohn (2006) have elaborated on SCFA interconversions on the basis of thermodynamic principles. These principles need better integration with relative abundance of various microbes, the metabolic pathways expressed by them, and their various approaches to handling asynchronous carbohydrate supply. Those authors discussed that interconversion of SCFA is much more thermodynamically likely for acetate than the more highly reduced propionate.

Multiple approaches have been used to inhibit methanogens to suppress enteric methane emissions from ruminant livestock operations (Hristov et al., 2013). However, many of these efforts were oversimplified because of reputed interacting dietary factors such as increasing forage digestibility and ruminal passage rate (Janssen, 2010). That author presented evidence that increasing aqueous H_2_ concentration would be associated with increasing methanogen growth rate but also decrease acetate production by the fermentative microbes based on thermodynamic principles. He acknowledged that the ratio of acetate to methane is not constant, but rather depends on whether H_2_ or propionate is formed along with acetate to balance reducing equivalents.

Interconversions of lactate and the SCFA could potentially influence various mechanistic models being derived to associate SCFA stoichiometry and methane production. For example, whether butyrate is produced through butyryl kinase or butyryl coA-acetyl coA transferase can vary the production of the intermediate lactate. If lactate is increased, there should be less of the intermediate H_2_ being produced in the first place and the lactate subsequently being fermented to propionate. If expressing butyryl coA-acetyl coA transferase does indeed have an advantage, probably through balancing reducing equivalents and ATP synthesis (Louis and Flint, 2009), then it might also allow a more efficient bacterial growth.

## Conclusions

Microbial protein production is inefficient largely owing to maintenance functions, accumulation of reserve carbohydrate, and energy spilling. Reserve carbohydrate is accumulated primarily by protozoa and under even modest carbohydrate excesses, whereas energy spilling occurs under larger excesses. Future work needs to identify microbial groups and biochemical mechanisms for spilling. Interconversion of lactate and SCFA is another potential mechanism for microbes to better manage rates of catabolic and anabolic reactions or, conversely, might be associated with energy spilling.

Principles summarized in this review could improve prediction of microbial protein production by mechanistic models, guiding efforts to maximize efficiency of that production. Some models already represent energy spilling and reserve carbohydrate, but most model parameter values are simple constants or heuristic (not derived directly from data) (Dijkstra et al., 1992; Russell et al., 1992; Dijkstra, 1994; Baldwin, 1995; Hackmann and Spain, 2010). For example, most mechanistic models assume storage of reserve carbohydrate is a constant fraction of microbial biomass (Russell et al., 1992; Baldwin, 1995; Hackmann and Spain, 2010). This review suggests that in order to improve prediction of microbial protein, models need improved representation of energy spilling and reserve carbohydrate, and it also points out experimental data needed to achieve this better representation.

### Conflict of interest statement

The authors declare that the research was conducted in the absence of any commercial or financial relationships that could be construed as a potential conflict of interest.

## Supplementary material

Figure S1 **Bucket model of energy spilling**. The large bucket represents the main pool of ATP-equivalents available to cell functions (maintenance, growth, reserve carbohydrate, energy spilling). The smaller bucket represents pool of ATP-equivalents in reserve carbohydrate, which can be stored from and mobilized to the main pool by pumps. Modified from Russell (2002).

Figure S2 **Dynamics of reserve carbohydrate accumulation for mixed bacteria in the rumen**. Diet was fed to calves once daily. Ingredient composition of basal diet 1.15 kg/d hay and straw and 1.26 kg/d flaked maize. Crude protein content was 2.56% DM for basal diet and between 6.56 and 8.32% for basal diet supplemented with urea. Reserve carbohydrate (“α-dextran glucose”) was measured as glucose detected by ion-exchange chromatography after hydrolysis in 0.5 *N* H_2_SO_4_ (100°C, 4 h). Figure redrawn from McAllan and Smith (1974).

Figure S3 **Dynamics of carbohydrate accumulation for the protozoan *Dasytricha ruminantium* in the rumen**. Diet was fed to sheep. Ingredient composition was 0.6 kg cubed molassed sugar-beet pulp and 0.3 kg chopped hay. Beet pulp was fed at 07.00 h and chopped hay fed at 16.00 h. Carbohydrate was measured by the phenol-sulfuric acid method after hydrolysis in 1 *N* NaOH (100°C, 5 min). Data from Williams and Harfoot (1976).

Figure S4 **Equations for synthesis and degradation of glycogen**.

Figure S5 **Equations for cellodextrin synthesis and efflux**. Na^+^ transport out of the cell is assumed to require 1/3 ATP (Russell, 2002). Cellobiose synthesis is shown, but synthesis of longer cellodextrins is also observed. After Wells et al. (1995).

Figure S6 **Amino acid (AA) biosynthesis in various bacteria, primarily *Escherichia coli***. PEP, phosphoenolpyruvate; OAA, oxaloacetate; SAM, S-adenosyl methionine; and HMB, 2-hydroxy-4-(methylthio) butanoic acid. Redrawn from Morrison and Mackie (1997), with minor modifications (Paulus and Gray, 1967; Baldwin and Allison, 1983; Or-Rashid et al., 2001; Walker et al., 2005). Numerous reactions are combined in the solid arrows representing enzymatic reactions. Examples of feedback inhibition are indicated by (−) and dashed arrows were excerpted from studies with non-rumen bacteria, again primarily *E. coli* (Gottschalk, 1979; Kalcheva et al., 1994; Hindson, 2003; Lohkamp et al., 2004; Yang et al., 2005; Caldara et al., 2008; Ferla and Patrick, 2014). Most rumen bacteria lack a complete TCA cycle but can make α ketoglutarate by forward or backward reactions from OAA (Wallace et al., 1997).

Figure S7 **Butyrate formation by two groups of ruminal *Butyrivibrio* species as proposed by Diez-Gonzalez et al. (1999) and further elaborated on by Paillard et al. (2007). (A)** Butyrate formation by butyryl-CoA/acetate CoA transferase, phosphotransacetylase, and acetate kinase. **(B)** Butyrate formation by butyrate kinase. See those sources for enzymes. The group on the right expresses butyrate kinase but produces more acetate, consumes little acetate, and produces little lactate. This group fully biohydrogenates to stearate. The group on the left expresses butyryl coA-acetyl coA transferase, produces more butyrate and lactate and consumes more acetate. Dashed boxes denote carbon input, although glucose is generalized from disaccharide or other sugars entering the glycolysis pathway and even as a phosphorylated monosaccharide. ATP, adenosine triphosphate; CoA, coenzyme A; GTP, guanosine tri phosphate; OAA, oxaloacetate; P, phosphate; and PEP, phosphoenolpyruvate.
