# Supplementary figures and images for: Maximizing efficiency of rumen microbial protein production
Source: Front Microbiol. 2015 May 15;6:465. doi: 10.3389/fmicb.2015.00465 (PMC4432691; doi:10.3389/fmicb.2015.00465)

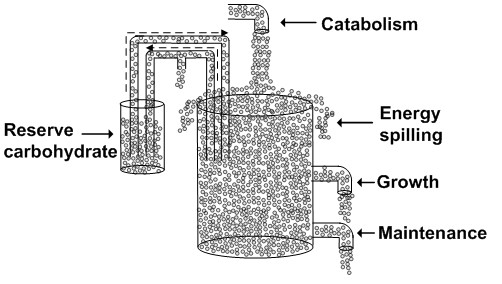

Supplement: Figure S1 — Bucket model of energy spilling. The large bucket represents the main pool of ATP-equivalents available to cell functions (maintenance, growth, reserve carbohydrate, energy spilling). The smaller bucket represents pool of ATP-equivalents in reserve carbohydrate, which can be stored from and mobilized to the main pool by pumps. Modified from Russell (2002). [file Image1.JPEG]

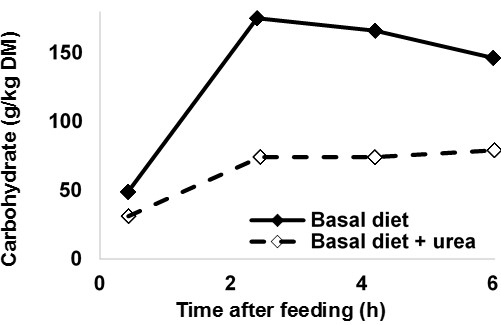

Supplement: Figure S2 — Dynamics of reserve carbohydrate accumulation for mixed bacteria in the rumen. Diet was fed to calves once daily. Ingredient composition of basal diet 1.15 kg/d hay and straw and 1.26 kg/d flaked maize. Crude protein content was 2.56% DM for basal diet and between 6.56 and 8.32% for basal diet supplemented with urea. Reserve carbohydrate (“α-dextran glucose”) was measured as glucose detected by ion-exchange chromatography after hydrolysis in 0.5 N H2SO4 (100°C, 4 h). Figure redrawn from McAllan and Smith (1974). [file Image2.JPEG]

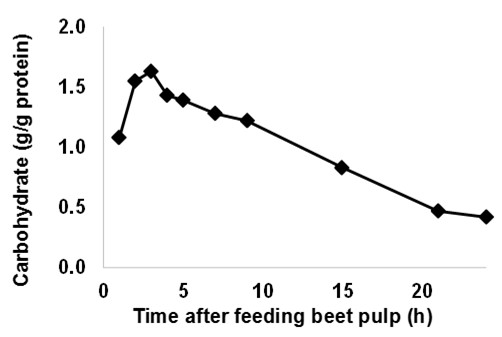

Supplement: Figure S3 — Dynamics of carbohydrate accumulation for the protozoan Dasytricha ruminantium in the rumen. Diet was fed to sheep. Ingredient composition was 0.6 kg cubed molassed sugar-beet pulp and 0.3 kg chopped hay. Beet pulp was fed at 07.00 h and chopped hay fed at 16.00 h. Carbohydrate was measured by the phenol-sulfuric acid method after hydrolysis in 1 N NaOH (100°C, 5 min). Data from Williams and Harfoot (1976). [file Image3.JPEG]

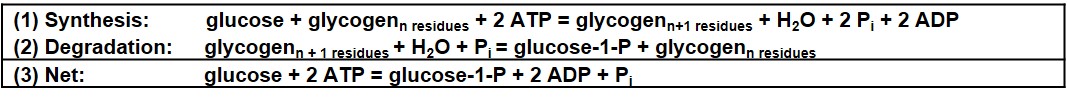

Supplement: Figure S4 — Equations for synthesis and degradation of glycogen. [file Image4.JPEG]

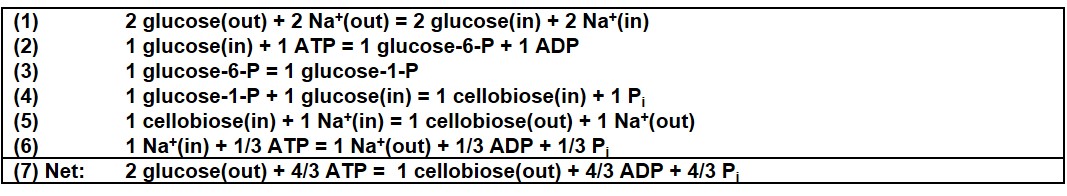

Supplement: Figure S5 — Equations for cellodextrin synthesis and efflux. Na+ transport out of the cell is assumed to require 1/3 ATP (Russell, 2002). Cellobiose synthesis is shown, but synthesis of longer cellodextrins is also observed. After Wells et al. (1995). [file Image5.JPEG]

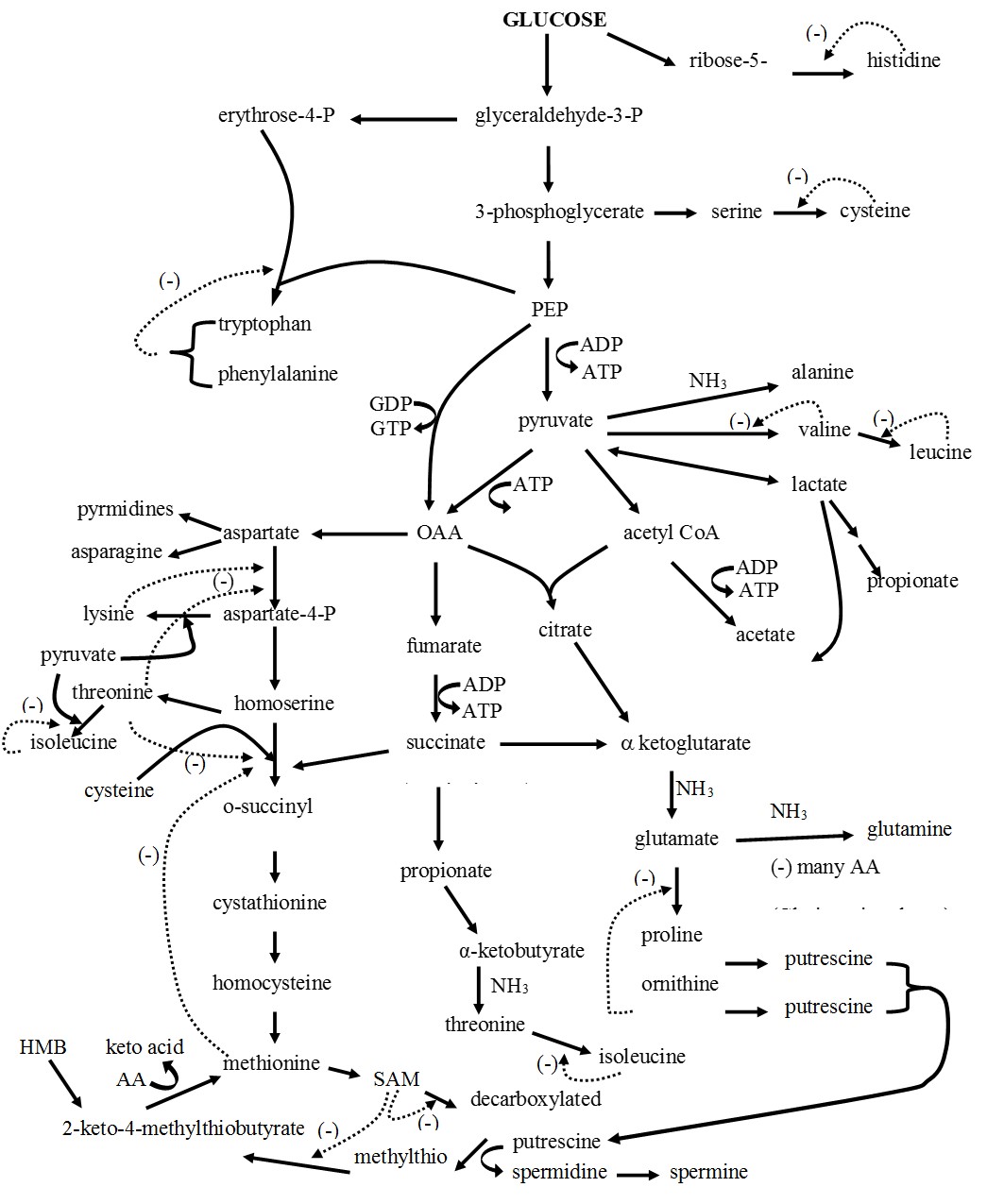

Supplement: Figure S6 — Amino acid (AA) biosynthesis in various bacteria, primarily Escherichia coli. PEP, phosphoenolpyruvate; OAA, oxaloacetate; SAM, S-adenosyl methionine; and HMB, 2-hydroxy-4-(methylthio) butanoic acid. Redrawn from Morrison and Mackie (1997), with minor modifications (Paulus and Gray, 1967; Baldwin and Allison, 1983; Or-Rashid et al., 2001; Walker et al., 2005). Numerous reactions are combined in the solid arrows representing enzymatic reactions. Examples of feedback inhibition are indicated by (−) and dashed arrows were excerpted from studies with non-rumen bacteria, again primarily E. coli (Gottschalk, 1979; Kalcheva et al., 1994; Hindson, 2003; Lohkamp et al., 2004; Yang et al., 2005; Caldara et al., 2008; Ferla and Patrick, 2014). Most rumen bacteria lack a complete TCA cycle but can make α ketoglutarate by forward or backward reactions from OAA (Wallace et al., 1997). [file Image6.JPEG]

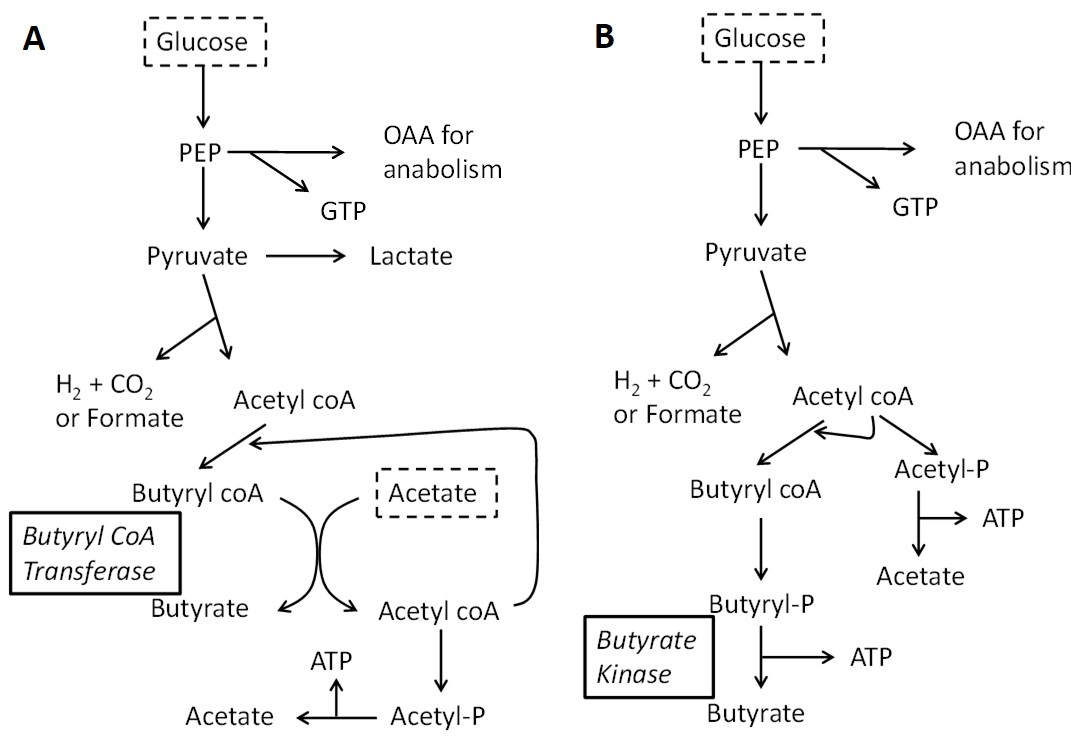

Supplement: Figure S7 — Butyrate formation by two groups of ruminal Butyrivibrio species as proposed by Diez-Gonzalez et al. (1999) and further elaborated on by Paillard et al. (2007). (A) Butyrate formation by butyryl-CoA/acetate CoA transferase, phosphotransacetylase, and acetate kinase. (B) Butyrate formation by butyrate kinase. See those sources for enzymes. The group on the right expresses butyrate kinase but produces more acetate, consumes little acetate, and produces little lactate. This group fully biohydrogenates to stearate. The group on the left expresses butyryl coA-acetyl coA transferase, produces more butyrate and lactate and consumes more acetate. Dashed boxes denote carbon input, although glucose is generalized from disaccharide or other sugars entering the glycolysis pathway and even as a phosphorylated monosaccharide. ATP, adenosine triphosphate; CoA, coenzyme A; GTP, guanosine tri phosphate; OAA, oxaloacetate; P, phosphate; and PEP, phosphoenolpyruvate. [file Image7.JPEG]
