# Supplementary material for: Maximizing efficiency of rumen microbial protein production
Source: Front Microbiol. 2015 May 15;6:465. doi: 10.3389/fmicb.2015.00465 (PMC4432691; doi:10.3389/fmicb.2015.00465)
Supplement: Supplementary file 8 [file Table1.DOCX]

Table S1 | Occurrence and characteristics of isolated reserve polysaccharide in rumen microbes

*^a^*

| Species | Monomer | Linkages | Chain length | Molecular weight (MDa) | Iodine staining*^b^* | Infrared spectrum*^b^* | | Reference |
| --- | --- | --- | --- | --- | --- | --- | --- | --- |
| **Bacteria** |  |  |  |  |  |  |  | |
| *“Bacteriodes amylogenes”* | Glucose | ND | ND | ND | Y | ND | | (Doetsch et al., 1957) |
| Eadie’s oval | Glucose | (α1→4) and (α1→6) | 11 | 4.3 | Y | Y | | (Orpin, 1973) |
| *Fibrobacter succinogenes* | Glucose | ND | ND | ND | Y | Y | | (Stewart et al., 1981; Gong and Forsberg, 1993) |
| *Megasphaera elsdenii* | Glucose | (α1→4) and (α1→6) | 9 | >0.2 | Y | Y | | (Cheng et al., 1973; Brown et al., 1975) |
| *Provetella ruminicola* | Glucose | (α1→4) and (α1→6) | 8 | >0.2 | ND | ND | | (Lou et al., 1997) |
| *Ruminococcus albus* | Glucose | ND | ND | ND | ND | ND | | (Hungate, 1963) |
|  | Glucose | (α1→4) and (α1→6) | ND | >0.2 | Y | Y | | (Cheng et al., 1977) |
| *Selenomonas ruminantium* | Glucose | ND | 12 | 0.5 to >2 | Y | ND | | (Wallace, 1980) |
|  | Glucose | (α1→4) and (α1→6) | 23.5 | 20 | Y | ND | | (Wakita and Hoshino, 1980) |
| Mixed streptococci | Glucose, trace mannose*^c^* | at least (α1→4) | ND | ND | Y | ND | | (Hobson and Mann, 1955) |
| **Fungi** |  |  |  |  |  |  | |  |
| *Neocallimastix* sp. LM-1 | Glucose | (α1→4) and (α1→6) | ND | ND | ND | ND | | (Phillips and Gordon, 1989) |
| **Protozoa** |  |  |  |  |  |  | |  |
| Mixed *Entodinium spp.* (chiefly *E*. *simplex* and *nanellum*) | Glucose | (α1→4) and (α1→6) | 22 to 25 | ND | Y | Y | | (Kamio et al., 1981) |
|  |  |  |  |  |  |  | |  |
| Mixed *Entodinium spp.* (chiefly *E*. *caudatum* and *simplex*) | Glucose | (α1→4) | 19 | ND | Y | ND | | (Eadie et al., 1963) |
| Mixed *Isotricha* and *Dasytricha* spp. | Glucose | (α1→4) and (α1→6) | 23 | 0.17 | Y | ND | | (Forsyth and Hirst, 1953) |
| **Mixed** |  |  |  |  |  |  | |  |
| Mixed rumen microbes | Glucose | (α1→4) and (α1→6) | ND | ND | Y | ND | | (Hackmann et al., 2013) |

*^a^*ND = not determined, Y = yes

*^b^*Characteristic of glycogen.

*^c^*Possible trace rhamnose

**References**

Brown, R.G., Lindberg, B., and Cheng, K.J. (1975). Characterization of a reserve glucan from *Megasphaera elsdenii*. *Can J Microbiol* 21**,** 1657-1659.

Cheng, K.J., Brown, R.G., and Costerton, J.W. (1977). Characterization of a cytoplasmic reserve glucan from *Ruminococcus albus*. *Appl Environ Microbiol* 33**,** 718-724.

Cheng, K.J., Hironaka, R., Roberts, D., and Costerton, J. (1973). Cytoplasmic glycogen inclusions in cells of anaerobic gram-negative rumen bacteria. *Can J Microbiol* 19**,** 1501-1506.

Doetsch, R.N., Howard, B.H., Mann, S.O., and Oxford, A.E. (1957). Physiological factors in the production of an iodophilic polysaccharide from pentose by a sheep rumen bacterium. *J Gen Microbiol* 16**,** 157-168.

Eadie, J., Manners, D., and Stark, J. (1963). The molecular structure of a reserve polysaccharide from *Entodinium caudatum*. *Biochem. J* 89**,** 91.

Forsyth, G., and Hirst, E. (1953). Protozoal polysaccharides. Structure of the polysaccharide produced by the holotrich ciliates present in sheep's rumen. *J Chem Soc***,** 2132-2135.

Gong, J., and Forsberg, C.W. (1993). Separation of outer and cytoplasmic membranes of Fibrobacter succinogenes and membrane and glycogen granule locations of glycanases and cellobiase. *J Bacteriol* 175**,** 6810-6821.

Hackmann, T.J., Keyser, B.L., and Firkins, J.L. (2013). Evaluation of methods to detect changes in reserve carbohydrate for mixed rumen microbes. *J Microbiol Methods* 93**,** 284-291.

Hobson, P., and Mann, S. (1955). Some factors affecting the formation of iodophilic poly-saccharide in group D streptococci from the rumen. *J Gen Microbiol* 13**,** 420-435.

Hungate, R.E. (1963). Polysaccharide storage and growth efficiency in *Ruminococcus albus*. *J Bacteriol* 86**,** 848-854.

Kamio, Y., Terawaki, Y., Nakajima, T., and Matsuda, K. (1981). Structure of glycogen produced by *Selenomonas ruminantium*. *Agric Biol Chem* 45.

Lou, J., Dawson, K.A., and Strobel, H.J. (1997). Glycogen formation by the ruminal bacterium *Prevotella ruminicola*. *Appl Environ Microbiol* 63**,** 1483-1488.

Orpin, C. (1973). The intracellular polysaccharide of the rumen bacterium Eadie's oval. *Arch Microbiol* 90**,** 247-254.

Phillips, M.W., and Gordon, G.L. (1989). Growth characteristics on cellobiose of three different anaerobic fungi isolated from the ovine rumen. *Appl Environ Microbiol* 55**,** 1695-1702.

Stewart, C.S., Paniagua, C., Dinsdale, D., Cheng, K.J., and Garrow, S.H. (1981). Selective isolation and characteristics of *Bacteriodes succinogenes* from the rumen of a cow. *Appl Environ Microbiol* 41**,** 504-510.

Wakita, M., and Hoshino, S. (1980). Physicochemical properties of a reserve polysaccharide from sheep rumen ciliates genus *Entodinium*. *Comp Biochem Physiol B Comp Biochem* 65**,** 571-574.

Wallace, R.J. (1980). Cytoplasmic reserve polysaccharide of *Selenomonas ruminantium*. *Appl Environ Microbiol* 39**,** 630-634.
